# Pure Oats as Part of the Canadian Gluten-Free Diet in Celiac Disease: The Need to Revisit the Issue

**DOI:** 10.1155/2016/1576360

**Published:** 2016-04-14

**Authors:** M. Cristina P. de Souza, Marie-Eve Deschênes, Suzanne Laurencelle, Patrick Godet, Claude C. Roy, Idriss Djilali-Saiah

**Affiliations:** ^1^Fondation Québécoise de la Maladie Cœliaque (FQMC), Montréal, QC, Canada H2J 3E6; ^2^Clinique de Gastroentérologie, Centre Hospitalier Lasalle, Lasalle, QC, Canada H8N 1X7; ^3^Service de Gastroentérologie, Hépatologie et Nutrition, CHU Sainte-Justine, Montréal, QC, Canada H3T 1C5

## Abstract

The question about recommending pure, noncontaminated oats as part of the gluten-free diet of patients with celiac disease remains controversial. This might be due to gluten cross contamination and to the possible immunogenicity of some oat cultivars. In view of this controversy, a review of the scientific literature was conducted to highlight the latest findings published between 2008 and 2014 to examine the current knowledge on oats safety and celiac disease in Europe and North America. Results showed that regular oats consumed in Canada are largely contaminated. Overall, the consumption of pure oats has been generally considered to be safe for adults and children. However, it appears that some oat cultivars may trigger an immune response in sensitive individuals. Therefore, further long-term studies on the impact of consumption of oats identifying the cultivar(s) constitute an important step forward for drawing final recommendations. Furthermore, a closer and more accurate monitoring of the dietary intake of noncontaminated oats would be paramount to better determine what its actual contribution in the gluten-free diet of adults and children with celiac disease are in order to draw sound recommendations on the safety of pure oats as part of the gluten-free diet.

## 1. Introduction

The gluten-free diet is the sole life-long treatment for individuals suffering from celiac disease (CD), an autoimmune enteropathy precipitated by dietary gluten. The restrictive diet consists of eliminating wheat, barley, and rye and other gluten-containing products which contain the toxic proteins prolamins. As an alternative cereal, the incorporation of oats in the gluten-free diet increases its nutritional value and variety. However, a small portion of individuals with CD has been shown to be sensitive to pure oats trough both external contamination by gluten and the presence of avenin, a type of prolamin which may be immunogenic [1]. A short review of the literature, from 2008 to 2014, regarding the safety of pure oats added to gluten-free diets consumed by individuals with celiac disease, was conducted to supplement those done previously and to further investigate the innocuity of pure oats [2]. Our primary objective was to gather the latest scientific findings obtained in North America and Europe, published after the 2007 Health Canada's position statement on the safety of oats as part of the gluten-free diet treatment of CD. The Health Canada report concluded that the majority of adults and children diagnosed with CD could tolerate moderate amounts of pure oats and that inclusion of pure oats into the gluten-free diet should be done after consultation with health professionals following complete resolution of the disease with a strict gluten-free diet [3]. Our review examined both extrinsic contamination and intrinsic immunogenicity of oat cultivars in an attempt to produce guidelines for the consumption of pure oats to individuals on a gluten-free diet in Canada. We included studies performed in adults and children/adolescents and review articles looking at oats consumption including recent research on oat varieties and their possible immunogenicity.

## 2. Methods

**Figure 1:**
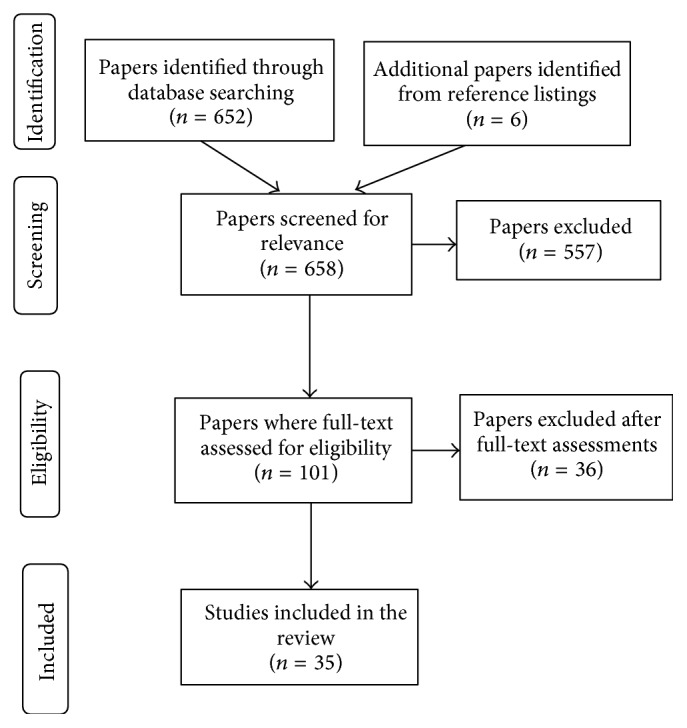
PRISMA Flow Diagram used in this review.

The search strategy used was MEDLINE via PubMed database with the following search terms: “oats,” “gluten-free diet,” “celiac disease,” and “avenin.” Articles were drawn from peer-reviewed journals, conference papers, systematic reviews, and government official reports. This brief literature review concentrated on the research published from January 2008 to December 2014 in attempt to identify studies published after the 2007 Health Canada's report on Celiac Disease and the Safety of Oats [3] and, hence, to gather the most recent scientific findings on the subject. It included publications in both English and French languages. A total of 652 papers were initially identified using the search terms previously described, and six suitable articles were also obtained by searching the reference lists of the identified articles, leaving a total of 658 articles. Thirty-five articles were finally included in this paper after careful screening and assessment, according to inclusion criteria previously described, following the PRISMA 2009 Flow Diagram [4] depicted in Figure 1.

## 3. Results

Even though this review covered a rather limit time frame, some interesting findings were revealed by European research groups examining potential immunogenic oat varieties used in their food supply. In order to draw final guidelines as to the safe introduction of oats in the gluten-free diets of Canadians suffering from CD, we revised both issues of intrinsic and extrinsic oat toxicity. There are numerous aspects to consider when comparing oat safety studies, particularly when comparing different biological and immunological parameter to assess oats toxicity. Another important compounding factor is the dietary information on the consumption of oats. Intake amounts and frequency are paramount and precise information are often lacking, not to mention the cultivars used in the production of pure oats in Canada.

### 3.1. Clinical Trials and Possible Avenin Immunogenicity in Celiac Disease

As far as the safety of oats in adults was concerned, six clinical trials were identified during this timeframe. These studies looked at a variety of clinical and laboratory parameters including levels of IgA antibodies to oats avenin, wheat gliadin, tissue transglutaminase, and endomysium; duodenal biopsies with histopathology and description of the villous pattern; gastrointestinal symptoms; and intraepithelial lymphocytosis among others to assess immunological response and presence of inflammation in vitro. Five were conducted in Europe [5–9] and one in Canada [10], and according to their results, five out of six studies proved that consumption of pure and noncontaminated oats was safe [5–7, 9, 10]. In one cross-sectional study, however, Tuire et al. found a persistent intraepithelial lymphocytosis in 56% of the celiac patients consuming pure oats, in spite of complete total intestinal mucosa recovery, with no reports of gastrointestinal symptoms and no signs of malabsorption [8]. Intake level and varieties of oats were not reported, and according to the authors, more studies were warranted to investigate, in a long-term fashion, any possible negative impact of oats on the intestinal mucosa focusing on intraepithelial lymphocytosis in celiac disease. In the Canadian study, fifteen subjects undertook a challenge test to evaluate the safety of certified pure oats produced under the guidelines proposed by the Canadian Celiac Association. The goal was to provide scientific evidence for the safety of oats consumption in North America. Participants were given 350 g of oats packed in bags over a 12-week period. They were mostly asymptomatic during the study period, with no significant differences in the biochemistry or histological parameters, suggesting that intake of pure oats was safe for this small group of subjects [10]. Overall, these studies differed in many aspects, namely, study design, number of subjects, time period, and clinical and biological parameters used, precluding us to make general conclusions or definitive statements. Furthermore, there were a disparity and lack of information on the dietary component when assessing oats consumption, such as precise amount of oats intake (before and during the study), the source of oats or oat-based foods, and the cultivar(s) used in some studies, which are all vital information when assessing the safety of oats in gluten-free diets.

When looking at the safety of pure oats consumption in gluten-free diets during childhood and adolescence, all five studies were conducted in Europe [11, 13, 18–20]. Three showed no toxicity induced by oats [11, 13, 20]. A randomized controlled trial in Finland investigated oats toxicity in 23 children with CD during a 2 yr period. Dietary intake was assessed by a registered dietitian using interviews and four-day food records. Subjects were randomly placed in two groups: oat challenge (13 children; median oat intake 45 g daily) and gluten challenge (10 children; median oat intake 41 g daily). Results showed no significant differences in immune activation and relapse of the CD, demonstrated by small intestinal mucosal morphology and detection of transglutaminase 2-specific IgA-deposits at the mucosal level, in both groups, except for two patients on the oats group [11]. A double-blind, randomized, placebo controlled multicenter study conducted in Italy, looked at gastrointestinal symptoms, serological markers (IgA anti-transglutaminase antibodies), and intestinal permeability (IP) in 306 children consuming medium to high intake of oats for 15 months. Even though accurate food diaries were used, the amount of oats ingested by older children was reported to be up to 40 g daily. Preliminary results indicated absence of changes in IP and gastrointestinal symptoms [13]. A long-term descriptive study looked at the compliance of gluten-free diets in 316 children and adolescents with reports of gastrointestinal and other symptoms in Sweden. About 89% of the participants had been consuming oats in their diets. Food questionnaires were elaborated by registered dietitians at three paediatric centers, after pilot testing prior to the investigation. The inclusion of oats was well tolerated by the majority of subjects. In this study, it should be noted that 81.9% consumed pure oats and 13.1% regular oats and 13.1% did not know what type of oats they consumed. Even though interesting results were obtained on dietary compliance, adverse effects were reported in the study questionnaires limiting the possibility of making comparison with the other studies [20].

Two other Swedish studies investigated the effect of pure uncontaminated oats consumption on different markers to assess the immune status and inflammation of younger subjects with CD [18, 19]. In a double-blind, randomized trial, Sjöberg et al. used expression levels of mRNAs for 22 immune effector molecules in paired biopsies of 28 children to assess their immune status and mucosal inflammation. The major finding was that intestinal immune status was not normalized in a substantial number of children in the oat group [18]. Tjellström et al. looked at the impact of pure oat consumption on the function of the microflora gut using fecal short-chain fatty acids (SCFA), as a marker to assess microbiome metabolism. A total of 71 CD children participated in the study. The daily consumption of oats recommended was 25 to 50 g but the reported mean was of 18 g in the oat group. Intestinal inflammation was detected in both the oat and the placebo groups. Children receiving oats had significantly higher levels of total SCFA throughout the 12-month period, indicative of an altered gut microflora function with possible presence of inflammation [19].

Contrary to the majority of results on oat consumption showing no adverse effects in adults and in children suffering from CD previously presented, it has been well established that some individuals with CD may react to pure, noncontaminated oat varieties. Avenin, the dietary protein prolamin found in oats, is thought to be responsible for immunological reactions observed in some celiac patients consuming pure oats [1]. Based on this assumption, a number of European studies examined the potential toxic effects of different oats in coeliac patients, which may vary according to their cultivars. Four studies using in vitro models of CD have identified some oat cultivars as immunogenic triggers [12, 15–17]. Comino et al. identified three immunogenic cultivars (OM719, OH727, and OF720) out of nine by examining T-cell proliferation and alpha-interferon release. Furthermore, the study showed the utility of the specific G12-antibody based ELISA technique in identifying different potentially toxic oat cultivars for patients with CD, due to an affinity of this antibody to the 33-mer peptide found in several oat varieties [17]. With the idea that some peptide fragments found in the gluten-like avenin protein in oats may be responsible for triggering immune responses, Mujico et al. investigated the amino acid sequences in 26 oat varieties obtained from the Netherlands. Avenin toxic peptides or epitopes, in pure oat varieties, which can potentially have adverse effect for some individuals with CD have been deciphered. The authors pointed out the importance of choosing and breeding safe cultivars, as a possible measure for inclusion of oats into gluten-free diets [15]. A Spanish in vitro study demonstrated a direct correlation between the immune-toxicity of five oat varieties with specific peptides and their protein sequences. The differences in the peptide sequences of various cultivars could explain the degree of their toxicity in celiac patients [16]. Out of three oat cultivars, Silano et al. found out that while the varieties* A. Potenza *and* A. Irina *did not induce the gliadin-induced transglutaminase-2 (dependent events in cell lines and in vitro model of CD)* A. Nave* did induce those mediated events. Therefore, their findings clearly indicated that distinctive differences in immunological response and inflammation were triggered by oat cultivars [12]. In Italy, Maglio et al. investigating biological and immunological characteristics of two cultivars, namely,* Avena Genziana* and* A. Potenza, *did not find in vitro evidence of avenin toxicity in the biopsies of 22 celiac subjects (age range: 2–57 years old) in remission [14].

Table 1 summarizes fifteen studies on safety of oats in CD comprised of clinical trials, analytical and in vivo studies, including five looking specifically at different oat cultivars and their possible immunogenicity in CD. Subjects from all but five studies [6, 12, 15–17] were following gluten-free diets for at least one year. Since the primary objective of this brief review was to gather the latest findings in Europe and North America, studies were categorized by country. Except for the Canadian study, all were conducted in Europe, indicating that additional studies are needed to evaluate the effect of oats consumed in Canada, especially long-term, which is paramount to determine the overall safety of oats for individuals suffering from CD. The duration of clinical studies varied from 12 weeks in Canada to eight years in Finland, and the majority was around 12 months not reflecting the impact of long-term oat consumption. Even though 87% of the studies used pure oats, there was a wide variation in daily oat consumption by adults, between 20 and 100 g, and by children, 20–59 g.

In 2011, Ballabio et al. studied thirty-six oat cultivars at the molecular level including avenin cross-reactivity. Although their results demonstrated that oats are considered safe for CD patients, the authors pointed out that selecting the most appropriate cultivar is critical due the great variation in gluten-like immunoreactivity amongst different varieties [21]. In addition, Halbmaryr-Jech and Hammer successfully developed and characterized a new sandwich monoclonal G-12 ELISA assay capable of detecting 33-mer from alpha-gliadin, the major peptide for triggering the gluten immune response in CD [22]. This technique was used for gluten analysis of a number of foods and beverages purchased at local supermarkets. Overall, very promising results on the gluten levels were obtained in all food samples, including gluten-free oats with a detection level of less than 1 ppm. The frequency of avenin epitopes which can be detected by T-cells from patients with CD was examined in thirteen* Avena* species by cloning avenin genes [23]. Results showed that all species presented two avenin-specific epitopes which could more likely explain T-cell responses in CD patients consuming pure oats.

### 3.2. Gluten Cross-Contamination: External Toxicity

In an attempt to examine the magnitude of gluten contamination from wheat, barley, or rye, Hernando et al. analyzed a total of 109 commercial oat foods from Europe, US, and Canada, and 25 pure and noncontaminated oat varieties from Spain [24]. Gluten analysis was performed using different techniques including the sandwich R5 ELISA essay. Results showed large contamination in regular oat foods, with levels exceeding 200 ppm, but very low levels of gluten, that is, below 3 ppm in pure uncontaminated oats. In 2008, a Canadian study designed to examine gluten contamination in cereal foods revealed that eight out of 12 commercial oat food products studied were contaminated with wheat and barley: two of them were labeled as gluten-free [25]. The authors emphasized the problem of misleading food labels as an important issue in the food choices of individuals on gluten-free diets. Later on, two analytical studies were also conducted in Canada to examine the degree of gluten contamination of the commercial oat supply. A large selection of 133 oat samples, consisting of packaged oat products, was collected from retail stores in five provinces (NF, PE, QC, ON, and BC) at two time points and analyzed by the RIDASCREEN R-7001 gliadin ELISA [26]. Only 9 samples (6.7%) were found to contain levels below 20 mg/kg, that is, the threshold adopted by the international food standards body Codex Alimentarius Commission for gluten-free foods; three had undetectable levels and six ranged between 5 and 20 mg/kg levels. The remaining 124 (93.2%) samples ranged from 21 to 3800 mg/kg, which indeed confirmed a contamination in the commercial oat supply by other grains. In spite of the fact that organic varieties had lower gluten levels than regular varieties, according to the authors, they could not be considered safe for gluten-free regimens. There was only one gluten-free sample which was confirmed indeed as gluten-free in this study. Furthermore, a number of different precautionary labeling statements were found among the oat products tested which may be very misleading and, namely, wheat-free claim, may contain traces of wheat, and so forth, and hence a cause for concern. Lastly, the authors pointed out that only certified pure oat products are suitable for consumption with gluten-free diets and that special requirements for all steps in the production of pure and uncontaminated oats in Canada, including growing, processing, analyzing, and labeling, should be controlled and followed. Moreover, certification for gluten-free products should be done by an independent certification organization accredited by the Standards Council of Canada. In 2013, a large analytical survey looked at gluten cross-contamination in 640 naturally gluten-free flours and starches sold in eight Canadian cities (Vancouver, Toronto, St. John, Calgary, Montreal, Halifax, Saskatoon, and Winnipeg) and via internet suppliers [27]. Sample selection was based on the mostly common products used in gluten-free diets of individuals with CD, between 2010 and 2012. A sampling technique, using the ten most popular Canadian and US gluten-free cookbooks, was created in order to generate a list of fifty-eight gluten-free flours and starches made from grains, nuts, and seeds. Consumption of these products was assessed by a descriptive frequency questionnaire in about ninety members of the Quebec Celiac Disease Foundation (*Fondation Québécoise de la Maladie Cœliaque*) and the Canadian Celiac Association. Based on the responses, commercially prepared bread flour mixes were reportedly commonly used. They were, therefore, also added to the sample pool. Results showed that 9.5% (61 out of 640) samples were contaminated with gluten at various levels (range of 5–7995 ppm). The authors concluded that products labeled gluten-free (i.e., with less than 20 ppm) were the safest to be consumed by Canadians with CD, while those not labeled as naturally gluten-free foods had a higher risk of contamination. A recent American study compared gluten levels in food products labeled gluten-free and certified gluten-free available on the US market [28]. This study attempted to gather data on gluten levels by testing 158 labeled gluten-free food products, including 46 that were certified gluten-free. Results showed that, overall, the majority of foods tested, that is, approximately 95%, had gluten levels <20 ppm, according to the gliadin sandwich R5 ELISA assay. Nevertheless, about 4% of the certified gluten-free samples contained gluten levels equal to or above 20 ppm. Labeled gluten-free samples of oats as grains and hot cereals (oats/mix) had gluten levels equal to or below 10 ppm.

Finally, eight review publications [29–36] were identified in the last seven years offering different opinions on the safety of oats in CD. Apparently, oat consumption among individuals suffering from CD still remains controversial and may not be completely harmless to all. The nutritional benefits of adding oats in the gluten restricted diets are well acknowledged and also scientific evidence indicates that, in Europe, a majority of CD patients can tolerate a moderate amount of pure noncontaminated oats in their diets. However, several factors precluded reaching a final conclusion on the safety of oats. There are several limitations of the current scientific literature related to oat consumption and of its impact on the health of individuals with CD. They include small number of subjects, lack of data on the cause of subject dropout, poor study designs, noncompliance of the study protocol by the participants, limited information on the long-term consumption of oats, the daily intake of oats, and the lack of identification of the oat cultivar(s) used.

## 4. Discussion

The purpose of this paper was not to produce an extensive literature review on the subject of oats safety and CD but rather to examine the latest findings published in the last seven years as supplemental information of the 2007 Health Canada's position statement on the safety of oats as part of the gluten-free diet treatment of CD. The information gathered in our review is essential for designing new Canadian guidelines and recommendations in the only life-time known treatment for individuals suffering from CD: a gluten-free diet.

This review showed that, in the last seven years, a number of classical and innovative markers are available to assess immune response and inflammation in CD. They comprise an array of clinical assays and in vitro laboratory tests, as well as very promising and highly sensitive techniques for the analysis of different protein fractions of prolamins contained in several cereals and food matrices. Studies in Finland, Ireland, Norway [4–8], and Canada [9] showed that adults suffering from CD were in clinical remission and did not present major adverse effects while consuming oats. Nevertheless, a persistent intraepithelial lymphocytosis was associated with oats consumption [8]. However, this was a cross-sectional study where subjects were classified in different groups (normal, inflammation, and atrophy), based on small intestinal biopsy findings, to look at differences between normal and inflammation groups in a number of variables. Lack of accurate data on oat intake and further clarification of the clinical significance of persistent lymphocytosis in adult patients with CD in a long-term setting were some major drawbacks in this study. Amongst randomized, controlled trials conducted in children, pure oat consumption did not trigger immune activation [11] or elicited changes in serological markers and intestinal permeability [13]. However, results from a randomized, double-blind multicenter intervention trial demonstrated that oat containing gluten-free diets did not normalize intestinal immune status response in a significant number of children [18] and it reduced fecal short-chain fatty acids concentration, which could indicate an ongoing inflammation [19]. These are important findings and more data on the small intestinal mucosal immune status and morphology; serological markers and intestinal permeability are warranted in new long-term studies particularly in North America, in both adults and children.

Potential immunotoxicity of oats has been demonstrated in European studies examining the safety of different oat cultivars on young volunteers with CD. These studies used different analytical techniques consisting of new antibody raised against the toxic fragment of avenin, such as monoclonal G12 based-ELISA techniques, R5 ELISA for gluten analysis, which may hamper comparisons amongst results. Another important aspect is determining which assays are more reliable to determine immunotoxicity, such as T-cell proliferation response and interferon *γ* release. Moreover, it is important to point out that some studies, looking at the safety of oats on gluten-free diets, lacked important information on the daily intake of oats consumed, their source, the cultivar(s) used, and degree of purity, therefore limiting their impact. For instances, clear information on purity of gluten-free samples is vital since many oat products are cross-contaminated and it is imperative to be able distinguish the source of toxicity, that is, gluten cross-contamination versus individual intrinsic toxicity of a particular cultivar. From a nutritional stand point, in order to safely advise about the amount of pure oats into gluten-free regimens, for both adults and children, additional studies examining intake amounts and type of gluten-free oat varieties are crucial to ensure a secure practice enhancing nutritional quality of the diet with a minimal health risk.

On the subject of external toxicity, all five studies identified in our review showed that gluten contamination exists in a variety of regular and gluten-free foods, including oat products, in Europe [24], USA [24, 28], and Canada [24–27]. Hence, gluten cross-contamination is a real problem in the Canadian oat supply and to a small extent in labeled gluten-free oats. Another relevant concern is regarding gluten-free precautionary labeling statements on oat products available in the Canadian market posing a serious problem by misleading consumers seeking gluten-free oats. A certification program is available and in place for all steps in the production of pure oats and can be done by independent certified laboratory accredited by the Standards Council of Canada. Therefore, only certified gluten-free oats (i.e., with less than 20 ppm) are recommended in the gluten-free diet in order to minimize the risk of external toxicity.

Furthermore, it is believed that variation in avenin epitopes, found in different oat varieties, can be identified as important contributors to some immunological reaction seen in patients with CD. These studies on avenin toxicity add another dimension to the presumed safety of pure oats and different oat cultivars may produce a substantial variation in gluten-like immunotoxicity indicating that a selection of an adequate variety for pure oats is crucial. The implications and long-term effects of these findings and information on the type of oat cultivars used to produce gluten-free oats in Canada are unknown.

In conclusion, most of the studies of oat safety in adults and children, including assessment of immune reactivity of different oat cultivars, have been conducted in Europe, where it seems that some countries possess a safe gluten-free oats production chain, specially dedicated for those suffering from CD. Further studies of the long-term impact of different oat cultivars in individuals suffering from CD on the immunogenicity of oats are called for, particularly in North America, in an attempt to clarify the issue of oat toxicity taking into consideration the type of oats cultivar used. Based on the latest scientific studies published, when introducing oats in the gluten-free diet of individuals with CD, only certified gluten-free oats should be allowed since cross-contamination is a real problem in commercially oats and oat-based foods in Canada. Large scale, randomized, double-blind trials, with well-documented amounts of daily oat intakes, GI symptom reports due to an increase in dietary fiber, and information on the specific cultivar(s) used, would be a great asset to ascertain a possible intrinsic toxicity of certified gluten-free oats manufactured in Canada, as potential dietary triggering factors. Results of long-term studies are urgently needed and would greatly help to (1) make appropriate comparisons with findings obtained from the European studies; (2) learn more about the oat varieties used in Canada for producing certified gluten-free oats; (3) study avenin toxic-peptides or epitopes that might have adverse effect for some individuals with CD and the mechanisms for triggering immune response; (4) gather reliable information on intake of oats; and (5) draw final recommendations on the inclusion of certified pure oats in strict gluten-free regimens.

## Figures and Tables

**Table 1:** Clinical trials and analytical and in vivo studies published between 2008 and 2014 on oats safety and celiac disease.

Country	Author reference	Year	Studies		Subjects		Oats			
			Type	Duration	Age group	Number	Consumption	Purity	Cultivar info	Toxicity
Canada	[10]	2011	Clinical	12 wk	Adults	15	350 g/wk	Yes	No	No
Finland	[9]	2013	CSFUS^1^	8 yr	Adults	106	20 g/d	?	No	No
	[8]	2012	Cross-sectional	N/A	Adults	177	?	?	No	Possible
	[6]	2010	RCS^2^	12 mo	Adults	31	100 g/d	Yes	No	No
	[11]	2009	RCT^3^	2 yr	Children	23	24–59 g/d	Yes	No	No
Ireland	[7]	2012	Clinical	1 yr	Adults	46	50 g/d	Yes	No	No
Italy	[12]	2014	In vitro	N/A	Children	3	N/A	Yes	Nave	Yes
	[13]	2013	DBRPCMT^4^	15 mo	Children	171	up to 40 g/d	Yes	No	No
	[14]	2011	In vitro	N/A	Children/adults	30	N/A	Yes	Yes	No
Netherlands	[15]	2011	In vivo	N/A	?	1	N/A	Yes	Yes	Yes
Norway	[5]	2008	Cross-sectional	N/A	Adults	277	24 g/d	Yes	No	No
Spain	[16]	2012	Analytical/in vivo	N/A	Children	14	N/A	Yes	OM719	Yes
	[17]	2011	In vivo	N/A	Children/adolescents	15	N/A	Yes	OM719, OH727, OF720	Yes
Sweden	[18]	2014	DBRMT^5^	1 yr	Children	28	20 g/d	Yes	No	Yes
	[19]	2014	DBRMT^5^	1 yr	Children	71	25–50 g/d	Yes	No	Yes

^1^CSFUS: cross-sectional follow-up study; ^2^RCS: randomized crossover study; ^3^RCT: randomized controlled trial;

^4^DBRPCMT: double-blind, randomized, placebo-controlled multicenter trial; ^5^DBRMIT: double-blind, randomized, multicenter trial.
